# Development of new powdery mildew resistant lines in garden pea (*Pisum sativum* L.) using induced mutagenesis and validation of resistance for the *er1* and *er2* gene through molecular markers

**DOI:** 10.3389/fpls.2024.1501661

**Published:** 2025-01-28

**Authors:** Akhilesh Sharma, Devinder Kumar Banyal, Vinod Janardan Dhole, Bansuli, Ranbir Singh Rana, Rajesh Kumar, Prabhat Kumar, Nimit Kumar, Srishti, Arshia Prashar, Vivek Singh, Anoushka Sharma

**Affiliations:** ^1^ Department of Vegetable Science and Floriculture, Chaudhary Sarwan Kumar Himachal Pradesh Krishi Vishvavidyalaya, Palampur, India; ^2^ Department of Plant Pathology, Chaudhary Sarwan Kumar Himachal Pradesh Krishi Vishvavidyalaya, Palampur, India; ^3^ Modular Lab, A Block, Nuclear Agriculture and Biotechnology Division (NABTD), Bhabha Atomic Research Centre, Mumbai, India; ^4^ Center for Geoinformatics Research and Technology, Chaudhary Sarwan Kumar Himachal Pradesh Krishi Vishvavidyalaya, Palampur, India; ^5^ Indian Council of Agricultural Research, Indian Institute of Vegetable Research, Varanasi, India; ^6^ Horticulture Commissioner, Ministry of Agriculture and Farmers Welfare, Government of India, New Delhi, India; ^7^ Department of Genetics and Plant Breeding, Chaudhary Sarwan Kumar Himachal Pradesh Krishi Vishvavidyalaya, Palampur, India

**Keywords:** mutagenesis, garden pea, *Erysiphe pisi* Syd, resistance, screening, er genes, molecular markers

## Abstract

Powdery mildew (PM) caused by *Erysiphie pisi* Syd. is the most devastating disease of pea, affecting fresh pea production as well as the quality of the marketable harvest worldwide. The efforts were made to develop PM-resistant mutants of popular pea varieties “Lincoln” and “Azad P-1” through induced mutations by following gamma irradiation (300, 400, 500, and 600 Gy) and chemical mutagenesis, i.e., ethyl methane sulfonate (EMS) (0.3% and 0.4%). The screening of 13,868 M_2_ progenies at Kukumseri (summer season) followed by M_3_ generation at Palampur (winter season) resulted in the isolation of six putative PM-resistant mutants. The rigorous evaluation of these progenies under *in vivo* (field screening) and *in vitro* (artificial screening under greenhouse conditions and using the detached leaf assay method) conditions over the years resulted in the isolation of three PM-resistant mutants, viz., L-40-1014, L-0.3-139, and AP-0.3-129. SSR markers “PSMPSAD60 d” and “PSMPA5 c” linked to the *er-1* gene indicated the presence of the “*er1*” gene in the mutant L-0.3-139 while the *er-2* gene-linked SCAR marker “ScX171400” and SSR marker “AD141” indicated the probability of the “*er-2*” gene in mutant L-40-1014. The known markers linked to PM resistance genes could not be validated in the mutant AP-0.3-129, suggested to identify new markers linked to PM resistance. These PM-resistant mutants can be promising candidates as the new source of resistance for future pea breeding programs.

## 1 Introduction

Garden pea (*Pisum sativum* L.) is an important leguminous crop worldwide belonging to the family *Fabaceae* and is cultivated for its fresh-shelled green seeds. The use of green shelled seeds in canned, frozen, or dehydrated products signifies its coveted position in the processing industry (Sharma et al., 2022). It is a rich source of nutrients such as proteins, vitamins, minerals, and lysine (an essential amino acid lacking in cereals), and its consumption, therefore, help to maintain human health (Sharma et al., 2020). In addition, fresh pea pods are exceptionally good sources of folic acid, vitamin C, vitamin K, and β-sitosterol (Rana et al., 2021). Its nutritional advantages are furthermore supported by its antidiabetic, antibacterial, antifungal, anti-inflammatory, anti-hypercholesterolemic, and antioxidant properties (Rungruangmaitree and Jiraungkoorskul, 2017). Apart from this, pea makes a substantial contribution in sustainable agriculture by improving soil health on account of its ability to fix atmospheric nitrogen and fits well in different crop rotation/cropping sequences, being a short duration crop.

Garden pea is grown commercially during winter season while the northwestern Himalayan region of India provides conditions for year-round cultivation as an off-season crop, i.e., in low and mid hills during winter season, and in high hills during summer season. India is the second-largest producer of fresh peas worldwide after China. Globally, pea occupies an area of 2.59 million ha with a production of 20.53 million tonnes (Anonymous, 2021). India occupies an area of 608.96 thousand ha with a production of 6631.29 thousand metric tonnes and average productivity of 10.89 metric tonnes ha^−1^ (Anonymous, 2024).

The major objectives for garden pea improvement are the development of high yielding varieties with desirable pod characteristics, i.e., long and dark green pods containing 8–10 seeds, sweet, and have high shelling besides carrying resistance to pests and diseases (Rana et al., 2021). Among the pea diseases, powdery mildew (PM; *Erysiphie pisi* Syd.) is the most devastating, causing very heavy yield losses to the extent of 25%–50% and thereby hampers the productivity and quality of produce (Fondevilla and Rubiales, 2012; Rana et al., 2023). The yield losses and green pod quality degradation are more conspicuous in the varieties of garden pea. In India, consumers prefer sweet, long, and dark green pods of garden pea that put Azad P-1 as the most preferred choice among different varieties, though it is highly susceptible to PM disease (Sharma et al., 2013). Inversely, PM-resistant varieties are generally carrying medium-sized pods with light-green color. It is, therefore, imperative to develop PM resistance varieties keeping in view the consumers’ preference for pod attributes without compromising yield. Genetic control is the most effective approach that is eco-friendly, cost-effective, and healthy that keeps green pods free from fungicide residues (Sharma et al., 2010).

The PM-resistant pea varieties can be developed through hybridization by involving the available resistance sources in the germplasm, which broadly indicated monogenic recessive inheritance (Sharma, 2003). The recessive “*er1*” gene imparts complete resistance against PM by restricting its penetration in the host and, therefore, “Mexique 4” and “S143” possessing “*er1”* gene were used extensively in a pea breeding program (Janila and Sharma, 2004; Ek et al., 2005). Another recessive gene, “*er2*”, was identified in “JI 2480”, which imparts resistance through post-penetration cell death (Fondevilla et al., 2006) and was also involved in PM resistance breeding. Apart from recessive genes, the dominant “*Er3*” gene was identified in *Pisum fulvum*, which is not temperature-dependent for its expression like “*er2*” (Fondevilla et al., 2007a; Bobkov and Selikhova, 2021). All the three resistance sources have been identified in pulse-type pea, and hence, their use as a donor for PM resistance is associated with traits like tall growth habit, and small and yellowish green pods with a high starch content that are undesirable in garden pea. Therefore, the pea varieties that are developed by involving the existing PM resistance sources lack consumers’ acceptance due to the light green/yellowish green and medium-sized pods. The regular use of these resistance sources to breed PM-resistant varieties also resulted in the narrow genetic base of modern cultivars along with compromised pod quality (Pandey et al., 2022).

The mutation breeding to some extent may help to resolve these issues besides help to identify new sources of resistance by rectifying a single undesirable trait while keeping all the other desirable traits intact as it does not involve hybridization (Bhat et al., 2001). The development of the “*mlo*” gene in 1942, conferring resistance to all forms of PM in barley, is a significant illustration of induced mutation (Dreiseitl, 2024). The application of induced mutation is the most appropriate in garden pea to create genetic variability because of its low chromosomal count (2n = 14) and short life cycle (Kalapchieva and Tomlekova, 2016). Pereira and Leitao (2010) identified and isolated two PM resistance mutants in pea. The natural loss of function of the PM-susceptible gene “*PsMLO1*” was identified as “*er1*” resistance gene that encouraged the mutation breeding for PM resistance in pea (Humphry et al., 2011; Pavan et al., 2011). Therefore, the present study was carried out for the development of PM-resistant mutants in popular pea varieties, viz., “Lincoln” and “Azad P-1” using physical (gamma irradiation) and chemical [ethyl methane sulfonate (EMS)] mutagenesis followed by their evaluation and screening under *in vivo* and *in vitro* conditions along with validation for PM-resistant genes “er1” and “er2” using known markers linked to PM resistance genes.

## 2 Materials and methods

### 2.1 Experimental material

Experimental material consisted of genetically pure seed of two popular varieties of garden pea, viz., Azad P-1 and Lincoln. Both the varieties are high yielding vis-a-vis possessing green and medium-sized pods containing eight seeds with a sweet taste but are highly susceptible to PM disease (**Table 1**).

**Table 1:** Salient features of popular pea varieties Azad P-1 and Lincoln.

Trait	Name of variety	
	Azad P-1	Lincoln
Days to first picking	125–135	140–145
Plant height (cm)	90–95	80–85
Pod length (cm)	8–10	7–9
Number of seeds pod^−1^	6–8	6–8
Average pod weight (g)	4–5	4–4.5
Shelling percent	45–48	42–45
TSS (°Brix)	15–17	15–17
Pod yield plant^−1^	60–70	60–65
PM reaction	Highly susceptible	Highly susceptible

#### 2.1.1 Mutagenic treatments

##### 2.1.1.1 Gamma ray

Four sets of 5,000 seeds of both the varieties with a uniform size and 10%–12% moisture content were irradiated with 300-, 400-, 500-, and 600-Gy doses of gamma rays in gamma cells (source: CO^60^) at Nuclear Research Laboratory, IARI, New Delhi.

##### 2.1.1.2 Ethyl methane sulfonate (EMS)

Another set of 2,500 pre-soaked seeds of each variety were treated with 0.3% and 0.4% EMS for 8 h at 30 ± 1°C with intermittent shaking in a gyratory shaker. The EMS-treated seeds were thoroughly washed in running water for 8 h to leach out excess EMS.

### 2.2 Experimental sites

The research project on the development of PM resistance lines through induced mutation in garden pea was carried out in Research Farms of Chaudhary Sarwan Kumar Himachal Pradesh Agricultural University at two diverse locations, i.e., Palampur and Kukumseri (Lahaul and Spiti). Palampur is situated at 1,290.8 m above mean sea level, 32°6′ N latitude and 76°3′ E longitude with a humid temperate climate (annual rainfall of 2,500 mm). On the other hand, Kukumseri Farm is located at an elevation of 2,672 m above mean sea level with latitudes of 31°44′ N and 76°41′ E, 360 km away from Palampur. It has a dry temperate climate with an annual precipitation of 250 mm and is the most suitable for shuttle breeding by advancing generations in off-season during summer vis-à-vis hot spot for screening genetic material for PM disease reaction.

### 2.3 M_1_ generation

Both irradiated and EMS-treated seeds were sown in the field at Vegetable Research Farm, Palampur to raise M_1_ generation during winter 2011–2012. All surviving M_1_ plants of the respective treatments of both the varieties were harvested individually that constitute 7,092 and 6,776 M_2_ progenies of Azad P-1 and Lincoln, respectively.

### 2.4 M_2_ generation

The plant-to-row M_2_ progenies derived from Azad P-1 (7,092) and Lincoln (6,776) were raised at Kukumseri to isolate PM disease-resistant mutants along with mutants for important morphological traits during summer 2012. The M_2_ population was carefully observed throughout the growth period and putative mutants were identified based on visual observations in comparison to susceptible parent varieties as control/check.

### 2.5 M_3_ generation

In the following winter 2012–2013, plant to row M_3_ progenies of two PM-resistant putative mutant of Lincoln vis-a-vis 142 mutant progenies identified for morphological traits of Lincoln (72) and Azad P-1 (70) were grown along with check varieties Lincoln and Azad P-1 at Palampur. The observations on PM disease incidence were recorded following the (0–4) scale of Mains and Dietz (1930) (**Table 2**) to identify and isolate PM-resistant mutants.

**Table 2:** Powdery mildew disease rating scale used for screening various mutagenized populations.

Disease score	Symptom description	Disease reaction
0	No trace of infection on any part	Completely resistant (HR)
1	Plant shows slight infection with roughly one in every four leaves infected, a fine coating of the powdery growth on the upper surface, the plant as a whole has green appearance, stems are free from infection, and plant size is normal.	Resistant (R)
2	Infection is moderate, nearly 50% of leaves are infected, with the upper ones being more severely infected, slight stem infection, and normal plant size.	Moderately susceptible (MS)
3	Nearly 75% of the foliage is infected, the whole plant appears to be covered with a white powdery coating, stems are also severely infected, and plant is slightly stunted.	Susceptible (S)
4	All the leaves of the plant as well as the stem are heavily coated with fungal growth, leaves turn pale green to yellow and start drying up, and the plant becomes conspicuous because of stunted growth.	Highly susceptible (HS)

### 2.6 Agronomic evaluation of powdery mildew resistant mutant progenies for important horticulture traits

The resistant mutant lines along with check varieties were evaluated for yield attributes by raising them in a randomized complete block design, replicated thrice at Kukumseri and Palampur from 2014 onwards. Each mutant with check was grown in two rows of 3 m length over the replications with an inter- and intra-row spacing of 45 cm and 7.5–10 cm, respectively. The observations were recorded on 10 randomly selected plants of each genotype in each replication for first flowering node, days to first picking, number of branches plants^−1^, internode length (cm), nodes plant^−1^, plant height (cm), pod length (cm), seeds pod^−1^ (number), shelling percentage, pods plant^−1^ (number), pod yield plant^−1^ (g), average pod weight (g), and harvest duration (days). The data on days to 50% flowering were recorded on a plot basis when 50% of the plants of the respective progenies had at least one flower opened.

The data were analyzed using analysis of variance (ANOVA) following Gomez and Gomez (1983) for the randomized complete block design and the significance of mean sum of squares for each trait was tested using the *F*-test at 5% level while critical difference (CD) using *t*-test was used for the comparison of means of different traits.

### 2.7 Screening of putative mutants for PM disease reaction

#### 2.7.1 Disease screening methods

**Figure 1:**
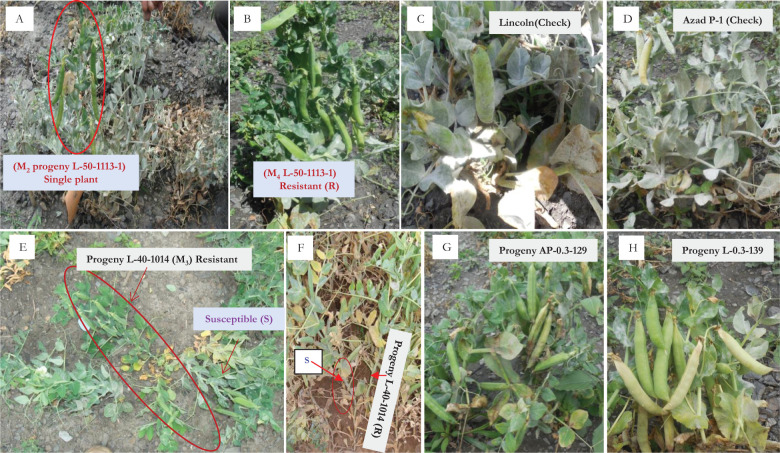
Comparative powdery mildew disease reaction. **(A, B)** L-50-1113 (M_2_ and M_4_), **(C)** Lincoln (highly susceptible), **(D)** Azad P-1 (highly susceptible reaction), **(E, F)** isolation of resistant mutant L-40-1014 (M_3_), **(G, H)** resistant mutants (AP-0.3-129 and L-0.3-139) under field conditions at Kukumseri.

**Figure 2:**
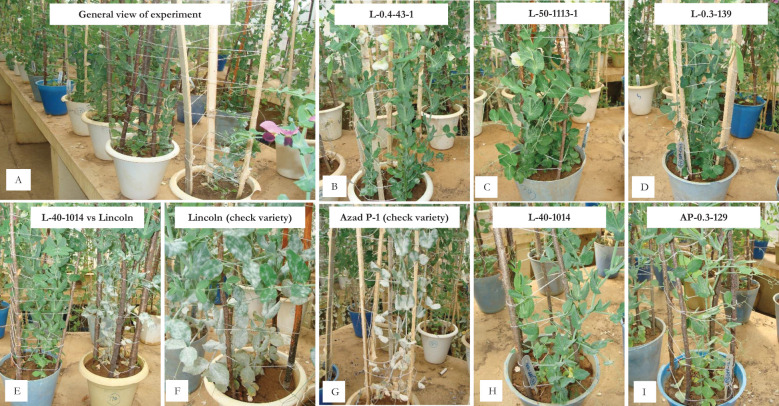
*In vitro* screening for powdery mildew disease reaction under nethouse conditions. **(A)** General view of the experiment; **(B)** L-0.4-43-1 (powdery growth on lower leaves); **(C, D)** L-50-1113-1 and L-03-139 (resistant reaction); **(E)** L-40-1014 (resistant) vs. Lincoln (susceptible); **(F, G)** susceptible check varieties Lincoln and Azad P-1; **(H, I)** L-40-1014 and AP-0.3-129 (resistant reaction).

The PM disease reaction in the mutant progenies was recorded by raising them under field (**Figure 1**) and *in vitro* conditions, i.e., in pots under naturally ventilated polyhouse conditions (**Figure 2**) at peak harvest of fresh pods (second/third picking) and at the seed maturity stage. The individual plants were categorized into different classes of disease severity following the (0–4) scale of Mains and Dietz (1930).

#### 2.7.2 Greenhouse screening

**Figure 3:**
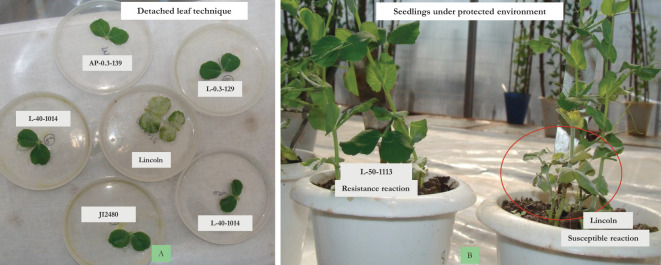
*In vitro* screening. **(A)** Detached leaf technique. **(B)** Isolation chamber with artificial inoculation.

To eliminate chances of disease escape, *in vitro* multiplied conidial inoculums of the disease were dusted on the plants with a camel hair brush for uniform development of disease infestation to facilitate effective screening of mutants for resistance under greenhouse conditions at Palampur (**Figure 3B**).

#### 2.7.3 *In vitro* screening following the detached leaf method

In addition to field and greenhouse screening, the detached leaf method was used to screen the resistant progenies under laboratory conditions (Banyal and Tyagi, 1999). Detached leaf assay of resistant mutants was conducted by floating the leaves of 3- to 4-week-old seedlings on the water solution in Petri dishes under controlled conditions in a plant growth chamber at Palampur (**Figure 3A**) by keeping temperature at 23 ± 2°C, 50%–70% humidity, and a photoperiod of 16 h light/8 h darkness. The disease inoculum was dusted on the leaves for further development. Observations on the disease development were studied for macroscopic and microscopic density of mycelia and sporulation at 9 days interval (Banyal et al., 2005).

#### 2.7.4 Confirmation of PM resistance mutants and their maintenance

The selection/screening process was carried out until stable lines are attained, i.e., uniform disease reaction across environments and other economic traits. The fixed lines (no variation in genetic makeup) with morphological attributes and PM disease resistance were maintained through evaluation under PM epiphytic conditions continuously until winter 2023–2024 to eliminate the chance of reverse mutation.

### 2.8 Molecular validation of mutant progenies for powdery mildew resistance genes

Eighteen primers (**Supplementary Table S1**), i.e., 11 markers (5 SSR and 6 SCAR) linked to *er1*, 4 markers (3 SSR and 1 SCAR) linked to *er2*, and 3 markers (2 SSR and 1 SCAR) linked to the *Er3* gene of PM resistance, were used for the validation of the resistant gene present in the respective mutants.

#### 2.8.1 Isolation, purification, and quantification of plant genomic DNA

The total genomic DNA was extracted from PM-resistant mutant lines, susceptible checks (Lincoln and Azad P-1), and *er1* and *er2* harboring lines (JI-1559 and JI-2480) followed by polymerase chain reaction (PCR) amplification using *er1*- and *er2*-linked markers. DNA was isolated from young leaf tissue by using the CTAB method (Murray and Thompson, 1980). The extracted DNA samples of all genotypes were loaded on 0.8% agarose gel (1 g/100 ml 1× TAE buffer) and run at 90 V for 40 min to determine the quality and quantity of DNA. Furthermore, the Nanodrop 2000c (Thermo Scientific) was used to verify the quantity and purity of the isolated DNA.

#### 2.8.2 Genomic DNA amplification in polymerase chain reaction

DNA amplification was carried out in a 12.5 µL reaction volume containing 20 ng of template DNA, 0.2 mM of each dNTP, 0.2 µM of each primer, 1.5 mM MgCl_2,_ 1× PCR buffer (10 mM Tris-HCl and 50 mM KCl, pH 8.3) and 1 U *Taq* polymerase. PCR amplification for all primers was carried out in a thermocycler (Make ProFlex) using initial denaturation at 94°C for 5 min followed by 39 cycles at 94°C for 30 s, 50°C–60°C for 30 s, 72°C for 1 min, and a final extension at 72°C for 5 min followed by rapid cooling at 4°C.

#### 2.8.3 Analysis of PCR product

Each PCR product (10 µL) was mixed with 3 µL of 6× gel loading dye (0.25% bromophenol blue and 40% sucrose) and amplified DNA fragments were separated on 4% agarose gel prepared in 1× TAE buffer and ethidium bromide (0.5 µg/mL). The gels were run at a constant voltage of 120 V for 1.5 h. The DNA profile was visualized by using the Gel Documentation system (Labnet, ENDURO™ GDS, Aplegen). The presence of appropriate size product of the markers linked to PM-resistant genes was recorded for putative mutant lines, susceptible checks, and *er1* and *er2* harboring lines by using 50 bp and a 1-kb ladder.

## 3 Results

### 3.1 Mutagenic effect on germination in M_1_ generation

Germination was reduced significantly in all the mutagenic treatments of both varieties (**Table 3**). Of the total 20,000 irradiated seeds each of Azad P-1 and Lincoln at 300, 400, 500, and 600 Gy that were sown to raise M_1_ population, a total of 4,853 and 5,570 individual plants that constitute only 24% and 28% of Lincoln and Azad P-1 were harvested, respectively, to raise plant-to-row M_2_ progenies. Similarly, 19% and 15% of plants were obtained from 10,000 EMS-treated seeds of the respective varieties at 0.3% and 0.4% EMS, i.e., 1,923 and 1,522 M_1_ plants. The resultant 13,868 M_1_ plant progenies of irradiated and EMS-treated seeds were harvested separately to raise M_2_ generation at Kukumseri.

**Table 3:** PM-resistant progenies isolated in M_2_ and M_3_ generations from different gamma rays irradiated and EMS-treated seeds of garden pea varieties Lincoln and Azad P-1.

Variety	Mutagenic treatment	Number of seeds treated	Germination % in M_1_	Number of M_2_ progenies raised at	M_2_ population size	Number of putative macro-mutants identified in M_2_	Number of PM-resistant mutant identified in M_2_	Number of PM-resistant mutant identified in M_3_
				Kukumseri during summer 2012				Palampur during winter 2012–2013
Lincoln	300 Gy γ-ray	5,000	22.62	1,131	3,619	10 (0.276)	–	–
	400 Gy γ-ray	5,000	20.28	1,039	2,389	12 (0.502)	–	1 (L-40-1014)
	500 Gy γ-ray	5,000	28.66	1,433	3,875	17(4.439)	1^*^ (0.0258) (L-50-1113-1)	–
	600 Gy γ-ray	5,000	25.00	1,250	3,439	10 (0.291)	–	
**Total (gamma ray)**		**20,000**	**24.21**	**4,843**	**13,322**	**49 (0.368)**	**1 (0.0075)**	**1**
Lincoln	0.3% EMS	2,500	45.84	1,146	2,642	14 (0.530)	–	1 (L-0.3-139)
	0.4% EMS	2,500	31.08	777	1,631	9 (0.552)	1^*^(0.0613) (L-0.4-43-1)	–
**Total (EMS treatments)**		**5,000**	**38.46**	**1,923**	**4,293**	**23 (0.536)**	**1 (0.023)**	**1**
Azad P-1	300 Gy γ-ray	5,000	28.38	1,419	4,402	10 (0.227)	–	–
	400 Gy γ-ray	5,000	29.54	1,477	4,189	9 (0.215)	–	–
	500 Gy γ-ray	5,000	27.02	1,351	1,553	20 (1.288)	–	–
	600 Gy γ-ray	5,000	26.46	1,323	3,406	20 (0.587)	–	–
**Total (gamma ray)**		**20,000**	**27.85**	**5,570**	**13,550**	**59 (0.435)**	**-**	**-**
Azad P-1	0.3% EMS	2,500	31.04	776	1,862	11(0.591)	–	2 (AP-0.3-439 and AP-0.3-129)
	0.4% EMS	2,500	29.84	746	1,838	**-**	–	–
**Total (EMS treatments)**		**5,000**	**30.44**	1,522	3,700	**11(0.297)**	–	2

* Powdery mildew symptoms appeared only on leaves, whereas stem and pod remained free from symptoms. * Values in parentheses are frequency of mutants per 100 M_2_ plants.

Bold values indicate total of each treatment in respective varieties (Lincoln and Azad P-1).

### 3.2 Generation of M_2_ and selection for powdery mildew resistant mutant progenies

Plant-to-row 13,868 M_2_ progenies were raised that resulted in the development of 34,845 M_2_ plants. Among this large population, only two putative mutant plants of “Lincoln” were isolated that carry a resistant reaction to PM disease and were designated as L-50-1113-1 (500 Gy) and L-0.4-43-1 (0.4% EMS) (**Table 3**; **Figure 1**). In addition, agronomically superior plants for various traits, viz., flower color, plant height, vigorous growth, and long pods, were also selected in M_2_ generation and were harvested separately to raise M_3_ generation.

### 3.3 Screening of powdery mildew disease resistant M_3_ progenies under polyhouse conditions (*in vitro*) and evaluation of morphological M_3_ progenies (*in vivo*)

Fortunately, out of 142 progenies carrying variable morphological traits, 4 progenies showed segregation for PM disease reaction under field conditions (*in vivo*) at Palampur during 2012–2013 and were designated as L-40-1014 (Lincoln at 400 Gy), L-0.3-139 (Lincoln at 0.3% EMS), AP-0.3-439, and AP-0.3-129 (Azad P-1 at 0.3% EMS) (**Table 3**). The M_3_ generation of L-50-1113-1 and L-0.4-43-1 was raised in progeny rows constituting 19 and individual plants of resistant progenies of both PM-resistant mutants and other desirable traits were raised at Palampur during winter 2012–2013. All 19 M_3_ progenies of L-50-1113-1 showed a resistant reaction whereas 23 progenies of L-0.4-43-1 showed susceptible reaction (**Table 4**). The resistant plants of these progenies were harvested individually and were further evaluated at Kukumseri during summer 2013 for validation of resistance.

**Table 4:** Powdery mildew disease reaction of putative mutants in M_3_ and M_4_ generation in polyhouse and field conditions during 2012–2013 at Palampur.

Name of variety	Mutant	Treatment	PM reaction in M_3_ generation Palampur (winter 2012–2013)		PM reaction in M_4_ generation at Kukumseri (summer 2013)
			Resistant	Susceptible	
Polyhouse screening					
Lincoln	L-50-1113-1	50 Gy γ-ray	19^*^	–	Resistant
	L-0.4-43-1	0.4% EMS	–	23	Susceptible
	Lincoln	Control	–	All	Susceptible
Field screening					
Lincoln	L-40-1014	40 Gy	4	7	Resistant
	L-0.3-139	0.3% EMS	3	6	Resistant
	Lincoln	Control	–	All	Susceptible
Azad P-1	AP-0.3-439	0.3% EMS	1	4	Resistant
	AP-0.3-129	0.3% EMS	3	4	Resistant
	Azad P-1	Control	–	All	Susceptible

* Powdery mildew symptoms appeared only on leaves, whereas stem and pod remained free from symptoms.

### 3.4 *In vivo* screening of resistant mutants in M_4_ and subsequent generations for powdery mildew reaction and yield attributes

All individual plant progenies of L-50-1113-1, L-40-1014-1, L-0.3-139, AP-0.3-439, and AP-0.3-129 carrying resistance to powdery disease reaction were evaluated separately in the following years along with pod and plant characters. The plant-to-row progenies of these five mutants that showed segregation or inferiority for pod attributes were discarded and only those with uniform pod and plant characteristic were selected for evaluating them for yield attributes, e.g., seven of L-50-1113 (-1, -2, -3, -4, -6, -8, and -10), two each of L-40-1014 (-1 and -6) and L-0.3-139 (-1 and -7), one of AP-0.3-439-1, and three of AP-0.3-129 (-1, -2, and -3) at Kukumseri during 2014 (**Table 5**; **Figure 1**). Majority of the plant-to-row progenies of the isolated mutants showed resistant reaction (1) with powdery growth in traces only on leaves at the end of vegetative growth while pods and stem remained free except AP-0.3-439-1 with a highly susceptible reaction (**Table 5**). The putative mutant progenies selected within the same group performed almost similar for PM and accordingly only one progeny in each set was retained with better performance for pod and plant attributes for further evaluation that constitute four mutants, namely, L-50-1113-1-6, L-40-1014-1-1, L-0.3-139-1-7, and AP-0.3-129-2. The mutant lines L-50-1113-1-6 and L-40-1014-1-1 recorded pod yields of 58.33 and 58.63 g plant^−1^, respectively, which were significantly better than check variety Lincoln (43.33 g) and Azad P-1 (40.67 g), and at par with Pb-89 (58.0 g).

**Table 5:** Performance of PM-resistant mutants in M_4_ generation for pod yield and related traits during summer 2014 at Kukumseri.

Progeny	Days to flowering	Pod length (cm)	Seeds/pod	Shelling (%)	Nodes/plant	Plant height (cm)	Average pod weight (g)	TSS (°Brix)	Pods/plant	Pod yield/plant	Powdery mildew reaction
L-50-1113-1-2	50	8.37	7.40	45.43	13.20	37.60	5.10	15.30	8.13	41.47	1
L-50-1113-1-3	50	9.03	7.43	47.33	14.67	33.53	5.18	15.70	10.48	54.33	1
L-50-1113-1-4	50	8.67	7.33	47.23	15.87	35.20	6.00	15.03	8.44	50.67	2
L-50-1113-1-6	52	8.83	7.60	48.43	15.00	31.73	4.95	15.82	11.79	58.33	1
L-50-1113-1-8	50	8.73	6.97	49.60	13.60	31.53	4.74	15.37	10.82	51.33	1
L-50-1113-1-10	51	8.73	6.97	49.63	14.80	32.33	5.15	15.23	11.00	56.67	1
L-40-1014-1-1	50	8.5	7.60	45.40	15.13	30.77	4.87	15.30	12.04	58.63	1
L-40-1014-1-7	49	8.87	6.90	47.43	13.53	33.00	4.71	14.17	8.06	38.00	1
L-0.3-139-1-1	50	8.9	7.20	50.30	12.67	34.00	5.13	15.47	9.09	46.67	1
L-0.3-139-1-7	51	9.2	7.13	46.43	11.93	31.53	5.35	15.13	8.84	47.33	1
AP-0.3-439-1	50	8.63	7.13	48.63	14.47	33.23	5.24	15.40	9.22	48.33	4
AP-0.3-129-1	51	8.13	6.73	45.97	12.47	33.47	4.95	15.37	6.94	34.33	4
AP-0.3-129-2	50	8.57	7.03	48.50	14.20	34.93	4.68	15.47	8.12	38.00	1
AP-0.3-129-3	50	7.92	7.00	49.93	13.73	34.20	4.96	15.13	7.05	35.00	1
Lincoln	53	8.57	7.10	48.70	14.47	32.53	5.91	15.13	7.03	43.33	4
Azad P-1	50	8.67	7.50	48.73	14.50	35.67	5.41	15.53	7.36	40.67	4
Pb-89	50	9.4	7.47	46.33	12.50	32.67	5.92	16.77	9.79	58.00	1
Range	49–53	7.92–9.2	6.73–7.47	45.4–50.3	12.5–15.87	30.77–37.6	4.71–6.0	14.17–16.77	6.94–12.04	34.33–58.63	1–4
Mean	50.41	8.69	7.21	47.88	13.93	33.41	5.19	15.37	9.07	47.12	1.76
CD (*p* ≤ 0.05)	2.68	0.66	0.55	NS	1.86	NS	0.45	0.77	2.00	8.49	-
Coefficient of variation (%)	3.17	4.78	4.73	5.11	8.19	10.47	6.32	3.05	14.25	11.70	–

NS, non-significant.

Furthermore, all these mutant progenies showed resistant reaction at Palampur during winter 2014–2015 under field conditions and using the detached leaf assay method (**Table 6**) along with a desirable performance for yield and its attributes, namely, pod length, seeds/pod, and shelling (%). Lines L-50-1113-1-6 and L-40-1014-1-1 produced pod yields of 93.20 and 94.25g plant^−1^, respectively, which were at par with check variety Lincoln and Azad P-1. These mutant progenies were finally designated as “L-50-1113”, “L-40-1014”, “L-0.3-139-1”, and “AP-0.3-129” for further evaluation/screening.

**Table 6:** Performance of PM-resistant mutants in M_5_ generation for pod yield, related traits, and disease reaction during winter 2014–2015 at Palampur.

Progeny	Days to flowering	Days to first picking	Pod length (cm)	Seeds/pod	Shelling (%)	Primary branches/plant	Internodal distance (cm)	Nodes/plant	Plant height (cm)	Average pod weight (g)	Pods/plant	Pod yield/plant	Powdery mildew reaction (*in vivo*)	Powdery mildew reaction (*detached leaf assay*)
L-50-1113-1-6	99.67	135.67	8.67	7.40	47.70	3.20	8.29	56.27	103.67	4.76	19.58	93.20	1	3
L-40-1014-1-1	95.33	135.33	8.83	7.23	48.83	2.87	7.03	53.53	99.37	4.56	20.67	94.25	1	1
L-0.3-139-1-7	97.33	134.00	10.40	6.90	45.72	2.80	6.10	51.67	62.27	4.60	14.35	65.83	1	2
AP-0.3-129-2	97.00	136.33	11.06	7.30	49.74	1.43	4.03	28.50	69.80	5.84	15.10	64.67	1	1
Lincoln (C)	101.67	143.67	9.56	7.33	43.80	2.67	7.20	42.13	82.80	4.41	19.63	86.67	4	4
Azad P-1(C)	98.67	135.00	10.22	7.13	46.10	2.93	6.23	45.53	93.67	4.81	17.43	84.17	4	4
Range	95.33–101.67	134–143.67	8.67–11.06	6.9–7.4	43.8–49.74	1.43–3.2	4.03–8.29	28.5–56.27	62.27–103.67	4.41–5.84	14.35–20.67	64.67–94.25	1–4	1–4
Mean	98.28	136.67	9.79	7.22	46.98	2.65	6.48	46.27	85.26	4.83	17.79	81.47	2.00	2.50
CD (*p* ≤ 0.05)	2.55	3.13	0.96	0.84	NS	0.93	0.84	7.34	7.95	0.54	2.97	11.21		
Coefficient of variation (%)	1.52	1.36	5.91	7.20	5.89	21.56	7.33	9.72	5.73	7.01	10.53	8.44		

^*^Powdery mildew reaction scale 0–4.

Where NS- non-significant.

### 3.5 Validation of powdery mildew reaction under *in vitro* conditions

The four resistant mutant progenies raised in pots under greenhouse conditions during 2014–2015 at Palampur by providing favorable conditions of temperature and humidity along with dusting of PM inoculum at the seedling stage showed resistance only in three mutants, i.e., L-40-1014, L-0.3-139-1, and AP-0.3-129, while L-50-1113 showed susceptible reaction. The further evaluation of these three resistant lines followed the same trend for disease reaction under *in vivo* and *in vitro* conditions over the years, i.e., at Kukumseri during 2017 and at Palampur for four consecutive years (2018–2019 to 2021–2022) under field conditions (**Table 7**), and net house/polyhouse (**Figure 2**) for six consecutive years (2018–2019 to 2023–2024) along with isolation chamber (**Figure 3**) at Palampur (2018–2019), which was further validated by the detached leaf assay method (**Table 8**, **Figure 3**). These three resistant lines L-40-1014, L-0.3-139, and AP-0.3-129 were further validated for the PM-resistant genes *er1, er2*, and *Er3* by using linked molecular markers.

**Table 7:** Reaction of mutant lines to PM disease under field conditions over 5 years.

Genotypes	2017	2018–2019	2019–2020	2020–2021	2021–2022	Overall	
	Kukumseri	Palampur				Disease score	Disease score
L-50-1113	3	3	3	4	3	3	S
L-40-1014	1	0	0	0	0	1	R
AP-0.3-129	1	1	1	1	1	1	R
L-0.3-139-1	1	1	1	1	1	1	R
Lincoln (C)	4	4	4	4	4	4	HS
Azad P-1 (C)	4	4	4	4	4	4	HS

R, resistant; MS, moderately susceptible; S, susceptible; HS, highly susceptible.

**Table 8:** Reaction of garden pea genotypes to PM disease under *in vitro* conditions over 6 years.

Genotypes	Naturally ventilated polyhouse							Detached leaf assay		Overall	
	Disease score						Disease reaction over the years	Disease score	Disease reaction	Disease score	Disease reaction
	2018–2019	2019–2020	2020–2021	2021–2022	2022–2023	2023–2024					
L-50-1113	3	3	3	3	4	4	S	3	S	3	S
L-40-1014	0	0	1	1	1	1	HR	0	HR	0	HR
AP-0.3-129	0	0	1	1	1	1	HR	0	HR	0	HR
L-0.3-139-1	1	1	1	1	1	1	R	1	R	1	R
Lincoln (C)	4	4	4	4	4	4	HS	4	S	4	HS
Azad P-1(C)	4	4	4	4	4	4	HS	4	S	4	HS

HR, highly resistant; R, resistant; MS, moderately susceptible; S, susceptible; HS, highly susceptible.

### 3.6 Validation of mutant lines for er1, er2, and Er3 genes by using linked molecular markers

**Figure 4:**
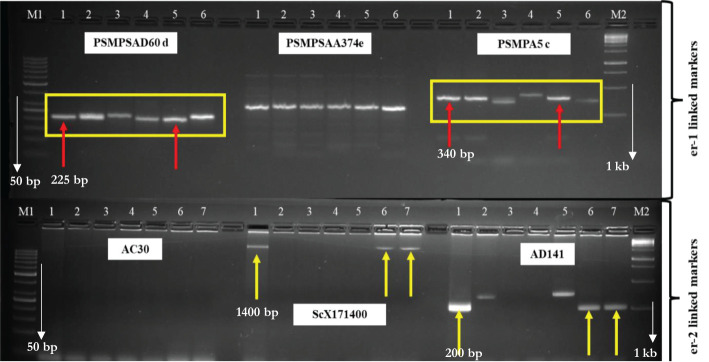
Agarose gel showing the presence/absence/polymorphism of amplification products of er-1-linked primers (PSMPSAD60 d, PSMPSAA374 e, and PSMPA5 c) and er-2-linked primers (AC30, ScX171400, and AD141) in the susceptible checks (Lincoln and Azad P-1), resistant checks (JI1559: source of er-1 gene, JI2480: source of er-2), and putative mutated lines (L-40-1014, L-0.3-139, and AP-0.3-129); M1 = 50 bp DNA Ladder, 1 = JI1559 in the first lane and JI2480 in the second lane, 2 = Azad P-1, 3 = AP-0.3-129, 4 = Lincoln, 5 = L-0.3-139, 6 = L-40-1014(1), 7 = L-40-1014(2), M2 = 1 Kb DNA Ladder.

Only two SSR markers “PSMPSAD60” and “PSMPA5” linked to the *er-1* gene differentiated the susceptible check, resistant checks, and mutants from one another (**Figure 4**). The primer “PSMPA5” linked to the *er1* gene showed that mutant L-0.3-139 derived from Lincoln had the same size amplification (340 bp) as that of resistant parent JI1559 indicating the possibility of resistance in L-0.3-139 due to the *er1* gene. The other SSR marker “PSMPSAD60” also validated resistance in L-0.3-139 due to the er1 gene as it showed the same size amplification (225 bp) as that of JI1559 (source of the *er1* gene) but the susceptible parent Azad P-1 also showed the band at the same position as in the resistant parent JI1559.

SCAR markerScX171400 linked to the *er2* gene showed the specific band at 1,400 bp in the resistant parent JI2480 (source of *er2*) and mutant L-40-1014 (**Figure 4**), indicating the possibility of the *er2* gene governing resistance in mutant L-40-1014. Similar results were recorded with the SSR primer AD141 as the resistant parent JI2480 and mutant L-40-1014 revealed the bands at the same position (200 bp) harboring the resistance governed by the *er2* gene. *Er3*-linked primers could not validate the presence in the mutants that may be due to the presence of recessive gene of PM resistance in the putative mutants.

## 4 Discussion

The artificial mutagenesis led to the introduction of innovative genetic variations that result in the development of new cultivars, functional description of genes, and the conception of gene–trait associations in many crops. Induced mutagenesis increases the frequency and range of mutations in the desired plant characteristics. The PM is one of the economically significant diseases of garden pea that has a marked effect on the quality and quantity of production. The management of disease through fungicides has severe consequences on human health and environment, and therefore, genetic resistance is ideal and sustainable. The management of PM in pea through resistance breeding has been the subject of several research investigations to date (Heringa et al., 1969; Tiwari et al., 1997; Fondevilla et al., 2007b; Katoch et al., 2010; Pavan et al., 2010; Sharma et al., 2013). Genetic resistance in plants has the advantage to boost productivity and handle the situation for a long period (Nigussie et al., 2008). Keeping in view the significance of pea and disease, we undertook the study on mutagenesis using both physical and chemical mutagens. The M_n_ generations were developed with an aim to isolate PM disease-resistant mutants.

We raised 13,868 M_2_ progenies consisting of 34,845 plants of two mutagenized populations of Lincoln and Azad P-1, both gamma irradiated and EMS treated for screening against PM disease infection. The screening was primarily undertaken at Highland Agricultural Research and Extension Centre, Kukumseri, Lahaul, and Spiti having a dry temperate climate and is situated in the northwestern Himalayas during the off-season in summer and we were able to isolate only two resistant plants that were designated as “L-50-1113-1” and “L-0.4-43-1”(**Table 3**; **Figure 1**). The very low frequency was obvious because the northwestern Himalayas are the hot spot for PM disease development on account of favorable environmental conditions for disease growth (Rana et al., 2013). They reported the evidence of existence of pathogenic virulence of *E. pisi* in the Himalayas because the cleistothecia (reproductive stage) of pathogen are usually established exclusively in the dry temperate zone. Furthermore, a more virulent race of pathogen identified as “Kukumseri” showed more disease pressure and severe illness development that substantiate the area as the hot spot for PM disease. Therefore, Kukumseri was preferred for M_2_ screening based on the high level of discrimination in the population. Simultaneously, 142 progenies were selected based on plant and pod characteristics. The 142 M_3_ plants were grown in plant-to row progenies for further selection at Palampur during 2012–2013 out of which four resistant progenies were isolated, i.e., L-40-1014, L-0.3-139, AP-0.3-439, and AP-0.3-129 (**Table 3**; **Figure 1**). The rigorous evaluation/screening of plant-to-row M_4_ and M_5_ progenies of these six mutants at Kukumseri and Palampur, respectively, resulted in the identification of four mutants, which were finally designated as “L-50-1113”, “L-40-1014”, “L-0.3-139-1”, and “AP-0.3-129”. The evaluation was continued over the years (2017 to 2024) under *in vivo* conditions at Palampur and Kukumseri (**Table 7**) along with *in vitro* conditions at Palampur (greenhouse/isolation chamber/natural ventilated polyhouse and detached leaf assay) (**Table 8**; **Figures 2**, **3**), which resulted in the identification of three mutants with a PM score of 1 (resistant), viz., “L-40-1014”, “L-0.3-139-1” and “AP-0.3-129”. *In vitro* testing with disease pathogen inoculation validates the presence of resistance in the cultivars. Line L-40-1014 also showed better performance for pod yield and other important attributes that can be utilized for commercial cultivation after multi-location testing.

In our current study, the resistant plants showed some patches of PM growth on the leaves at the end of the vegetation period especially at Kukumseri, suggesting some kind of quantitative expression of the resistance. Pereira and Leitao (2010) reported that this type of quantitative expression is misleading, and a simple, but rigorous screening of individual plants will permit its qualitative classification in clearly distinct classes, either resistant or susceptible. The strong qualitative expression of the resistance can be recorded at the end of the vegetative period where the disease infestation profoundly appears on pods, causing deterioration of pod quality and making them unfit for consumption. In contrast, the designated resistant plants showed no spots of infection on pods and resulted in unequivocally healthy pods. In other words, categorization of PM resistance can be established during the vegetation stage based on inclusive plant appearance and pod infection (Sharma, 2003). Based on multiple experiments on PM resistance, it was concluded that the resistant plants can be qualitatively distinguished based on infection on the stem, peduncles, and pods, while ignoring the fungal growth on the foliage whatever its intensity.

Three putative resistant lines L-40-1014, L-0.3-139-1, and AP-0.3-129 identified from the present study have the PM resistance potential and can be further used as a resistant source in pea breeding programs. These three putative resistant lines were further validated for the PM resistance gene by linked molecular markers. Only two *er1* and *er2* recessive genes and one dominant *Er-3* gene have been identified to provide resistance to *E. pisi* (Harland, 1948; Heringa et al., 1969; Fondevilla et al., 2007a). It was found that the bulk of *E. pisi*-resistant pea germplasm has the *er1* recessive gene, and it confers long-term broad-spectrum resistance to PM disease (Tiwari et al., 1997). Researchers found different markers linked to the resistance genes *er1* and *er2* and the markers were placed at varied genetic distances in different mapping populations. Ek et al. (2005) found five SSR markers (PSMPSA5, PSMPSAD51, PSMPSAD60, PSMPSAA374e, and PSMPSAA369) linked to the *er1* gene with PSMPSAD60 being the most closely associated marker, at 10.4 cM distance from the *er1* locus, and PSMPA5 c at 14.9 cM. They are flanked markers for the *er1* gene. These markers showed nearly the same size amplification in the mutant L-0.3-139 as that of the resistant parent JI1559, indicating the possibility of resistance to be governed by the *er1* gene. Pereira et al. (2010) identified two altered genes, *er1* mut1 and *er1* mut-2, by using ethyl nitrosourea mutagenesis, which leads to the development of PM-resistant pea mutants, and the SSR marker PSMPSA5 was found to be related to the *er1* mut-2 locus at a greater distance. Thus, our findings suggest that the mutation has affected the *er1* locus, which is frequently found in naturally occurring resistances for PM. It was also observed that mutations are more frequent in the *er1* locus as compared to the *er2* locus (Pereira and Leitao, 2010). The *er1* gene was placed on linkage group VI (Timmerman et al., 1994) and the *er2* gene was located on linkage group III (Katoch et al., 2010). Molecular markers found closely linked to the resistance locus will be beneficial for marker-assisted breeding that exploits the mutant gene to create new PM-resistant cultivars. SCAR marker ScX171400 (Katoch et al., 2010) linked to the *er2* gene clearly validated the *er2* gene in mutant L-40-1014 by showing the specific band of 1,400 bp in a resistant parent (JI 2480) and mutant L-40-1014. Similarly, the SSR marker AD141 at 9.3 cM linked to *er2* gene/allele (Gupta et al., 2023) also validated this gene in the resistant parent and mutant L-40-1014 by depicting a specific band of 200 bp.

Systemic acquired resistance (SAR) plays a significant role in the genetic processes of pathogen resistance, which is related to the expression of resistance genes and the induction of the hypersensitive reaction (Pavan et al., 2010; Martins et al., 2020). This mechanism of resistance helps plants to deal with the presence of nonspecific and opportunistic infections on a regular basis. Susceptibility genes (S-genes) are the genes that encode special target proteins. Loss-of-function mutations in S-genes frequently result in broad-spectrum resistance to a specific pathogen (Eckardt, 2002; Pavan et al., 2010). In the mutant AP-0.3-129, none of the gene is confirmed for the resistance but Azad P-1 susceptible check showed a band pattern like the *er1* gene. Thus, the resistance in the mutant AP-0.3-129 may be due to the loss-of-function mutations in S-genes. The gene *er1* has been widely employed in pea breeding, although having the same source of resistance may increase the incidence of new races and eventually leads to resistance breakdown. Combining many key genes from diverse sources into a variety can result in long-lasting resistance and the mutants identified in the present study can be promising candidates for the new source of resistance for future pea breeding improvement. This breeding method should be supplemented using more accurate PM resistance-linked molecular markers. The key disadvantage is that such markers are obtained from genomic areas that play no causal role in the development of resistance and may show a similar pattern in both susceptible and resistant genotypes. As a result, gene-based markers resulting from functional polymorphisms within resistance genes must be utilized to screen the germplasm for target genes. The specific use of molecular markers in the improvement of pea breeding accentuates the development of PM resistance new gene-specific markers. The progress of high-throughput next-generation sequencing (NGS) and genotyping technologies has resulted in a significant decrease in the cost for developing molecular markers, which paves the way for a marker-assisted breeding approach as a more wide-ranging, useful, efficient, and cost-effective method in the future.

## 5 Conclusions

The current study resulted in the isolation of three new PM disease resistance lines, viz., L-40-1014, L-0.3-139, and AP-0.3-129, following induced mutagenesis, and these were validated for PM-resistant genes *er1* and *er2* by using linked molecular markers. SSR markers PSMPSAD60 d and PSMPA5 c linked to the *er1* gene indicated the presence of this gene in the mutant L-0.3-139 while SCAR marker ScX171400 an SSR marker, AD141, linked to the *er2* gene clearly validated the *er-2* gene in mutant L-40-1014. The resistance in the mutant AP-0.3-129 was found to be governed by a different mechanism and may be due to the loss-of-function mutations in S-genes. Line L-40-1014 also showed promise for pod yield and other important pod and plant attributes. The identified mutants with PM resistance potential can be promising candidates as new sources of resistance for future pea breeding programs.

## Data Availability

The original contributions presented in the study are included in the article/**Supplementary Material**. Further inquiries can be directed to the corresponding author.
