# Supplementary material for: Development of new powdery mildew resistant lines in garden pea (Pisum sativum L.) using induced mutagenesis and validation of resistance for the er1 and er2 gene through molecular markers
Source: Front Plant Sci. 2025 Jan 28;15:1501661. doi: 10.3389/fpls.2024.1501661 (PMC11810882; doi:10.3389/fpls.2024.1501661)
Supplement: Supplementary file 1 [file Table1.docx]

**Table S1. List of primers used for molecular studies**

| S.No. | Primer | Gene/allele | Primers Forward Sequence | Primers Reverse Sequence |
| --- | --- | --- | --- | --- |
|  | PSMPSAD60 | er-1 | CTGAAGCACTTTTGACAACTAC | ATCATATAGCGACGAATACACC |
|  | PSMPSAA374e | er-1 | GTCAATATCTCCAATGGTAACG | GCATTTGTGTAGTTGTAATTTCAT |
|  | PSMPA5 | er-1 | GTAAAGCATAAGGGGATTCTCAT | CAGCTTTTAACTCATCTGACACA |
|  | PSMPSAA369 | er-1 | CCCTTCGCACACCATTCTA | AGTCGTTTTGGAGATCTGTTCA |
|  | PSMPSAD51 | er-1 | ATGAAGTAGGCATAGCGAAGAT | GATTAAATAAAGTTCGATGGCG |
|  | ScAH1R | er-1 | GATGGACCCCATCAAGTAC | GCCCCAACTTCATGTCTTG |
|  | ScOPO061100 | er-1 | CCCCATGTTAGAACCTTGCA | ACGGGAAGGTCTGACAGTAT |
|  | ScOPL13990 | er-1 | ACCGCCTGCTCTGATGTG | GCGCTGCTTAATCTCAGG |
|  | ScAGG.CAA125 | er-1 | GAATTCAGGAACATAGCTTC | CAAGCTAAAAGTCAGAAGAT |
|  | ScOPT16480 | er-1 | GGGCAGAATCAGCTGAGCTC | GAACAAGGAGAAGAAGAGG |
|  | ScOPX 04880 | er-1 | CCGCTACCGATGTTATGTTTG | CCGCTACCGAACTGGTTGGA |
|  | ScX171400 | er-2 | GGACCAAGCTCGGATCTTTC | GACACG GACCCAATGACATC |
|  | AD141 | er-2 | AATTTGAAAGAGGCGGATGTG | ACTTCTCTCCAACATCCAACGA |
|  | AC30 | er-2 | GCAGCAAGAGTGACGAAGTTATC | GCCTGACTACCACTTCTGCTG |
|  | AA-278 | er-2 | CCAAGAAAGGCTTATCAACAGG | TGCTTGTGTCAAGTGATCAGTG |
|  | ScW4637 | Er-3 | CAGAAGCGGATGAGGCGGA | CAGAAGCGGATACAGTACTAAC |
|  | AD61 | Er-3 | CTCATTCAATGATGATAATCCTA | ATGAGGTACTTGTGTGAGATAAA |
|  | AA349 | Er-3 | ACCATGAATCCCATATAGAGAG | GTTTGATCCCAATATCTTACCA |
